# Necrological

**Published:** 1888-03

**Authors:** 


### NECROLOGICAL.

DIED.—Dr. T. M. Pattillo, of Mount Vernon, January 24, 1888.

DR. S. A. OGLE, of Duffan's Mills, died January 26, 1888.

DR. JNO. BELL, of Dallas, died January 15, 1888.

DR. — HARDING, of Terrill, died February 8, 1888.

DR. J. J. DAVIS, of Atlanta, Texas, was killed by the accidental discharge of a gun, January 28, 1888.

DR. THOS. H. HOLLIS, of Nacogdoches, died on Friday, February 3, 1888, at his residence, of dilatation of the heart, complicated with cirrhosis of the liver; these diseases having come upon him gradually, and keeping him confined to his room for a month previous to his death.

He was born July 22, 1829, in the State of Tennessee. At an early age he came to San Augustine, Texas, where his parents resided till their death, and where he was educated. He graduated in Medicine at the University of Pennsylvania, in 1854. He began the practice of medicine in Anderson county, Texas, where he married Miss Katie Dumas, December 21, 1857.

He was a surgeon in the Confederate army, serving first east of the Mississippi, afterward west of it, being on duty at the battles of Shiloh, Mansfield and others, where he was noted for his daring but conservative operations. Returning to San Augustine after the war, he soon removed to the town of Nacogdoches, where he practiced many years; afterwards to Wills Point, thence to Elmo, and to Anson, in Jones county, and finally back to Nacogdoches, a few months before his death. His professional skill, especially in the direction of operative surgery, and his manly stand for the dignity of his profession, were his leading characteristics, and his reputation was not limited to his locality. He had a wide social as well as professional acquaintance. He was gifted with a strong mind and a high degree of courage, energy and vim. M.

